# Removal of Necrosed Bone by Irrigations with Weak Hydrochloric Acid

**Published:** 1887-09

**Authors:** Edmund Andrews

**Affiliations:** Professor of Clinical Surgery in Chicago Medical College, Surgeon of the Mercy Hospital, etc.


### Removal of Necrosed Bone by Irrigations with Weak Hydrochloric Acid.

Read in the Section on Surgery at the Thirty-Eighth Annual Meeting of the American Medical Association, June, 1887.

BY EDMUND ANDREWS, M.D., L.L.D.,

Professor of Clinical Surgery in Chicago Medical College, Surgeon of the Mercy Hospital, etc.

In almost all cases of necrosis the dead bone can be removed without operation by the simple process of daily irrigation with diluted hydrochloric acid. This is especially important in spinal necrosis and caries; but is also applicable to necrosis in other parts of the body.

Nearly all our authors are silent on this subject, for though Dupuytren and other older surgeons have spoken of brushing the cavity with strong sulphuric acid, they contemplated merely a caustic action to change the condition of the diseased living parts, and not the dissolving out of the sequestrum. Others, however, have proposed the solvent plan, but without gaining a serious hearing in surgical literature. Billroth, of Vienna, briefly alludes to the subject, but scouts the whole idea in the following strong but ill-considered lauguage: "Chemical solution of the sequestrum is not to be thought of. If you were daily to pour muriatic acid into the fistulous opening, it would affect the newly formed osseous tissue as much or more than it would the sequestrum, which would be very unfortunate, as it must replace the latter. Hence, the mechanical removal of the sequestrum is the only thing left."

In opposition to this high authority I wish to state as the result of actual experiments that, by a little ingenuity in the management of the irrigation, most sequestra can be dissolved out; and if the solution be properly prepared it does not affect the living bone, nor act upon the soft parts, except as a good antiseptic; neither does it cause any pain. In fact, by the addition of cocaine and morphine, it can be made anodyne.

So far as my investigations have yet gone, I find no solvent equal to a weak solution of hydrochloric acid. Sulphuric acid seems to limit its own action by precipitating the insoluble calcium sulphate in the Haversian canals, and blocking the further access of the acid. Thin laminæ of bone will lie in a dilution of this acid for days with only a slight degree of decalcification. Chromic acid is also inefficient, and stains the linen on which it happens to drip. Nitric acid decalcifies the bone rapidly, but I have not yet finished the experiments for ascertaining its practical usefulness.

The proper strength of the hydrochlóric solution varies with the tolerance of the patient, the object being to keep it just below the painful point. This will usually be found between one-fourth and one-sixteenth of the strength of the officinal *acidum hydrochloricum dilutum*, although it can be employed somewhat stronger by the addition of cocaine and morphine. The tolerance seems to be determined by the presence or absence of a good coat of granulations upon the living tissues. Directly after an operation the cut extremities of the nerves are painful on the application of any acid, however weak; but after a few days, when covered by granulations, they bear comparatively strong solutions without difficulty.

The length of time required will vary with the strength of the solution and the constancy of the irrigation. The following experiments were made outside the body to get an idea of the rapidity of the action of the acid. Fragments of actual sequestra which had been removed by operation were placed in bottles of acid of various strengths, and the time required for complete decalcification noted.

TABLE SHOWING THE TIME REQUIRED FOR DECALCIFYING SEQUESTRA IN WEAK HYDROCHLORIC ACID SOLUTIONS OUTSIDE THE BODY.

| Experim't | Strength of Solution. | Dimensions of Sequestra. | Time required for Decalcification |
|---|---|---|---|
| 1 | 1-16 the strength of officinal dilute hydrochloric acid | 4½ cm. by 1 cm. by 3 mm. | 142 hours |
| 2 | 1-10 do do | 3½ cm. by 14 mm. by 4 mm., very hard seq. from tibia. | 120 " |
| 3 | 1-10 do do | 4½ cm. by 28 mm. by 20 mm. Very spongy tuberculous trochanter. | 24 " |
| 4 | ⅛ do do | 9 cm. by 32 mm. by 5 mm. | 117 " |
| 5 | 1-12 do do | 38 mm. by 9 mm. by 4 mm. | 120 " |
| 6 | ⅛ do do | 2⅓ cm., very hard sequestr. | 128 " |
| 7 | ⅛ do do | 5½ cm. by 13 mm. by 1 cm. | 102 " |
| 8 | ⅙ do do | 8 cm. long, whole thickness of shaft of femur, diam. 2 cm. | 96 " |
| 9 | ¼ do do | 32 mm. by 13 mm. by 3 mm. | 102 " |
| 10 | ¼ do do | 64 mm. by 38 mm. | 54 " |
| 11 | ¼ do do | Block of cancellated bone 16 mm. diameter. | 78 " |
| 12 | ¼ do do | Sundry small splinters. | 54 " |

Average time of solution, about 95 hours.

These experiments show that old sequestra, which have laid long in putrid suppurating cavities, are not deprived of their gelatiniferous animal basis. In every instance the decalcified animal matter remained in full form, but, lying enclosed in granulations inside the body, it is rapidly absorbed, exactly like the decalcified bone drainage tubes when left in position.

The method of effecting the irrigation is important. If one simply pours a little acid into the fistula, as Billroth evidently understood the plan to be, he will accomplish nothing. It is necessary to irrigate the whole sequestrum either frequently or continuously. If possible one should have two openings, so situated that the fluid may run into one, and thence along the whole length of the sequestrum and out at the other. When necessary new openings must be made in suitable positions, and left pervious by drainage-tubes. The next step is to fill a half-gallon fountain syringe with the acid solution, and, hanging it by the bedside, to let it run very slowly through the diseased channel. If a "through and through" irrigation is not possible, we can often obtain the same result by taking a small flexible catheter, especially of the kind having a curve near the tip, and called by the French *sonde coudee.* By a little careful management this can often be carried to the farthest corner of the cavity, and by attaching it to the fountain syringe it will deliver the fluid beyond the sequestrum, and make it flow over it on its return outside the catheter. Whenever the sequestrum can be thoroughly irrigated its decalcification is a matter of absolute certainty within a moderate number of days. The hydrochloric solution is a perfect antiseptic of itself, and during its use no other is required.

After the solution is completed there remains the gelatiniferous animal matter of the bone, which is identical with the material of decalcified bone drainage-tubes, and like them is rapidly absorbed in antisepticized cavities. I have commenced to investigate the possibility of dissolving it out at once with pepsin, but have not finished that part of the subject.

Billroth raises the objection that the acid will dissolve the living bone as rapidly as the dead, or even more so. So plain a point as this of course can not fail to rise in every one's mind, but I am sure that great man spoke from mere theory, and not from observation or experience. Whatever one might fear in this respect, I find as a matter of experience that the weak solutions which I have used do not decalcify living bone. Apparently the granulations covering the living osseous tissue protect it from contact with the acid, except in spots denuded by ulceration, and even there the circulation of the alkaline blood in the Haversian canals, and the constant exudation of alkaline serum from all raw living surfaces, bone included, protect it from the action of such weak acid dilutions as I have hitherto used. At any rate, it is a positive fact that the sequestra are dissolved and the cavity heals without any perceptible injury to the living bone.

—*American Med. Assoc'n Journal.*

